# Identification and characterisation of SARS-CoV-2 and *Human alphaherpesvirus* 1 from a productive coinfection in a fatal COVID-19 case

**DOI:** 10.1590/0074-02760210176

**Published:** 2022-01-10

**Authors:** Alice Laschuk Herlinger, Fábio Luís Lima Monteiro, Mirela D’arc, Filipe Romero Rebello Moreira, Harrison James Westgarth, Rafael Mello Galliez, Diana Mariani, Luciana Jesus da Costa, Luiz Gonzaga Paula de Almeida, Carolina Moreira Voloch, Covid19-UFRJ Workgroup, Adriana Suely de Oliveira Melo, Renato Santana de Aguiar, André Felipe Andrade dos Santos, Terezinha Marta Pereira Pinto Castiñeiras, Ana Tereza Ribeiro de Vasconcelos, Esaú Custódio João, Claudia Caminha Escosteguy, Orlando da Costa Ferreira, Amilcar Tanuri, Luiza Mendonça Higa

**Affiliations:** 1 Universidade Federal do Rio de Janeiro, Instituto de Biologia, Departamento de Genética, Laboratório de Virologia Molecular, Rio de Janeiro, RJ, Brasil; 2 Universidade Federal do Rio de Janeiro, Instituto de Biologia, Departamento de Genética, Laboratório de Diversidade e Doenças Virais, Rio de Janeiro, RJ, Brasil; 3 Hospital Federal dos Servidores do Estado do Rio de Janeiro, Rio de Janeiro, RJ, Brasil; 4 Universidade Federal do Rio de Janeiro, Instituto de Microbiologia Paulo de Góes, Departamento de Virologia, Laboratório de Genética e Imunologia das Infecções Virais, Rio de Janeiro, RJ, Brasil; 5 Laboratório Nacional de Computação Científica, Laboratório de Bioinformática, Petrópolis, Brasil; 6 Universidade Federal do Rio de Janeiro, Rio de Janeiro, RJ, Brasil; 7 Instituto de Pesquisa Professor Amorim Neto, Campina Grande, PB, Brasil; 8 Universidade Federal de Minas Gerais, Instituto de Ciências Biológicas, Departamento de Genética, Ecologia e Evolução, Belo Horizonte, MG, Brasil; 9 Universidade Federal do Rio de Janeiro, Faculdade de Medicina, Departamento de Doenças Infecciosas e Parasitárias, Rio de Janeiro, RJ, Brasil

**Keywords:** SARS-CoV-2, COVID-19, HSV-1, coinfection, coronavirus

## Abstract

**BACKGROUND:**

During routine Coronavirus disease 2019 (COVID-19) diagnosis, an unusually high viral load was detected by reverse transcription real-time polymerase chain reaction (RT-qPCR) in a nasopharyngeal swab sample collected from a patient with respiratory and neurological symptoms who rapidly succumbed to the disease. Therefore we sought to characterise the infection.

**OBJECTIVES:**

We aimed to determine and characterise the etiological agent responsible for the poor outcome.

**METHODS:**

Classical virological methods, such as plaque assay and plaque reduction neutralisation test combined with amplicon-based sequencing, as well as a viral metagenomic approach, were performed to characterise the etiological agents of the infection.

**FINDINGS:**

Plaque assay revealed two distinct plaque phenotypes, suggesting either the presence of two severe acute respiratory syndrome coronavirus 2 (SARS-CoV-2) strains or a productive coinfection of two different species of virus. Amplicon-based sequencing did not support the presence of any SARS-CoV-2 genetic variants that would explain the high viral load and suggested the presence of a single SARS-CoV-2 strain. Nonetheless, the viral metagenomic analysis revealed that *Coronaviridae* and *Herpesviridae* were the predominant virus families within the sample. This finding was confirmed by a plaque reduction neutralisation test and PCR.

**MAIN CONCLUSIONS:**

We characterised a productive coinfection of SARS-CoV-2 and Herpes simplex virus 1 (HSV-1) in a patient with severe symptoms that succumbed to the disease. Although we cannot establish the causal relationship between the coinfection and the severity of the clinical case, this work serves as a warning for future studies focused on the interplay between SARS-CoV-2 and HSV-1 coinfection and COVID-19 severity.

In Brazil, severe acute respiratory syndrome coronavirus 2 (SARS-CoV-2) has spread widely, totaling more than 21 million cases and 596,122 deaths (as of October 1, 2021; https://covid19.who.int/). SARS-CoV-2 infections vary from asymptomatic cases to mild, moderate, and severe Coronavirus disease (COVID-19). In severe cases, apart from the acute respiratory syndrome, the central nervous system can be compromised, as encephalopathies have already been reported in the literature.^1^

The gold standard COVID-19 diagnosis relies on detecting viral nucleic acids through reverse transcription real-time polymerase chain reaction (RT-qPCR) from nasopharyngeal swab samples. Threshold cycle (Ct) values provide a semiquantitative measure of virus load, with SARS-CoV-2 Ct values from 17-24 considered as an indicator of a high viral load.^2^

In early May 2020, during the routine SARS-CoV-2 diagnostic, we noticed an unusually low Ct value (N1 Ct = 5) RT-qPCR result in a sample from a severe COVID-19 patient. Besides the typical COVID-19 symptoms, this patient also displayed neurological manifestations. Aiming to characterise the etiological agent that caused such a severe disease, we used classical virological methods combined with high-throughput sequencing (HTS) technologies. While no SARS-CoV-2 mutations associated with higher viral loads, like the ones reported for variants of concern or interest, were identified, we detected the presence of a productive coinfection of SARS-CoV-2 and *Human alphaherpesvirus* 1 - previously known as Herpes simplex virus 1 (HSV-1) - which might have contributed to the poor outcome observed.

## MATERIALS AND METHODS

*COVID-19 molecular diagnosis* - Total virus nucleic acids were extracted from nasopharyngeal swab specimens using the Maxwell^®^ 16 Viral Total Nucleic Acid Purification Kit (Promega). SARS-CoV-2 detection was performed by RT-qPCR using the 2019-nCoV CDC qPCR Probe Assay (IDT) and GoTaq Probe One-Step qPCR Assay (Promega) in an Applied Biosystem 7500 Real-Time PCR System.

*Viral load* - To calculate the viral load, a standard curve from the 2019-nCOV_N Positive Control (IDT) was constructed (1, 5, 10, 50, 100, 1,000, and 10,000 copies), and the sample was submitted to RT-qPCR targeting N1 and N2, as described for the COVID-19 molecular diagnosis. The RNA copy number was converted to a logarithmic scale and adjusted to linear models (N1: r^2^ =0.99, p < 0.001; N2: r^2^ = 0.934, p < 0.01) to estimate the SARS-CoV-2 RNA copy number in the sample. The analysis was performed in the R software.

*Subgenomic RNA* - The presence of subgenomic RNA was inferred by RT-qPCR with specific primers and probes for the E gene (FwsgRNAE: 5’-CGAT CTC TTG TAG ATC TGT TCT CTA AAC GAA CTT ATG TAC TC-3’; Gene E_Sarbeco_R2: ATA TTG CAG CAG TAC GCA CAC A-3’; E_Sarbeco_P1: 5’-FAM-ACA CTA GCC ATC CTT ACT GCG CTT CG-Iowa Black-3’), and the N gene (FWsgRNAN:5’-CGA TCT CTT GTA GAT CTG TTC TCT AAA CGA ACA AAT TAA AAT G-3’; 2019-nCoV_N1-R: 5’-TCT GGT TAC TGC CAG TTG AAT CTG-3’; 2019-nCoV_N1-P: 5’-FAM-ACC CCG CAT TAC GTT TGG TGG ACC-Iowa Black-3’), using the GoTaq Probe One-Step qPCR Assay (Promega) in a 7500 Real-Time PCR System (Applied Biosystems).

*Viral isolation* - For viral isolation, the nasopharyngeal swab sample (6439_SW_) was filtered through 0.22 µm membrane (Millipore) and diluted in DMEM (Gibco) supplemented with 1% penicillin/streptomycin. Two-fold serial dilutions (1:3 to 1:384) of sample 6439_SW_ were inoculated into Vero CCL-81™ (ATCC) seeded in 6-well plates (2 x 10^5^ cells/well). After 2 h, the inoculum was removed, and DMEM supplemented with 2% fetal bovine serum (FBS; Gibco) and 1% penicillin/streptomycin was added. Cells were monitored for cytopathic effect daily. Three days after inoculation, we performed blind passage by transferring 500 μL of conditioned media to new 6-well plates seeded with Vero cells at a density of 2 x 10^5^ cells/well. After three days, the conditioned medium was harvested, centrifuged at 300 × *g*, and sterile-filtered to remove cells and cellular debris. The sample was identified (6439_CM_), aliquoted, and stored at -80ºC for further analysis.

*Plaque assay* - To quantify infectious virus particles, a plaque assay was performed. Confluent monolayers of Vero cells were inoculated with ten-fold serial dilutions of filtered nasopharyngeal swab sample 6439_SW_ (1:3 - 1:300,000). After 1 h, the inoculum was removed, and cells were overlaid with a semisolid medium (alpha-MEM supplemented with 1% FBS and 1.25% carboxymethylcellulose). Cells were further incubated for three days and fixed with 4% formaldehyde. Cells were stained with 1% crystal violet in 20% ethanol for plaque visualisation. Titers were expressed as plaque-forming units (PFU) per milliliter.

*Amplicon-based SARS-CoV-2 HTS* - Amplicon-based complete SARS-CoV-2 genome sequencing was performed as previously described.^3^ Briefly, total RNA obtained from the swab sample (6439_SW_) was used for cDNA synthesis using the Superscript IV first-strand synthesis system (Thermo Fisher) prior to PCR using the IDT ARTIC nCoV-2019 V3 Panel and the Q5 High-Fidelity DNA Polymerase (New England Biolabs). PCR products were cleaned up using AmpureXP beads (Beckman Coulter) and quantified using Qubit dsDNA High Sensitivity assay (Thermo Fisher Scientific). Libraries were prepared using the TrueSeq DNA Nano kit (Illumina), and quality control was performed using the High Sensitivity D1000 ScreenTape Assay on a 4200 TapeStation system (Agilent, USA). HTS was performed in a MiSeq System with MiSeq Reagent Kit v3 (Illumina, USA) set to obtain 2×250 bp reads.

Prior to data analysis, raw read sequences were preprocessed as follows: quality control analysis was performed using the FastQC v0.11.4 (https://www.bioinformatics.babraham.ac.uk/projects/fastqc/); low quality reads (average quality < 25) were filtered using trimmomatic v0.39;^4^ cutadapt v2.1^5^ and clumpy v38.41 (https://sourceforge.net/projects/bbmap/) were used to remove optical duplicates in 5’ primer regions. The reference-based SARS-CoV-2 genome assembly, using the Wuhan-Hu-1 reference (NCBI accession number: NC_045512.2) and the variant analysis, was performed using the Geneious Prime (Geneious) software.

Virome Methodology

*Sample preparation* - To access the viral diversity, we performed an additional HTS using a metagenomic approach. For 6439_SW_, total nucleic acids were isolated directly from 100 μL of biological material using the QIAamp MinElute Virus Spin Kit (Qiagen) without any prior treatment. For the 6439_CM_ sample, 200 μL of conditioned medium was clarified by filtering through a 0.22 µm sterile filter (Millipore) to remove eukaryotic cell-sized particles and cellular debris. Total nucleic acids from the filtrate enriched in viral particles were isolated using the QIAamp MinElute Virus Spin Kit (Qiagen). For both samples, the extraction protocol followed the manufacturer’s instructions, with the following modifications: (i) the “Carrier RNA” was omitted from the AL Buffer; (ii) the protease was resuspended in AVE Buffer, instead of Protease Resuspension Buffer; (iii) the washing step with AW1 was suppressed; (iv) the final elution was performed with 20 μL of ultra-pure water. Thereafter, an RT-PCR reaction was performed with SuperScript^TM^ III First-Strand Synthesis System (Invitrogen) for first-strand cDNA synthesis from RNA viruses, using random primers, while preserving DNA viruses. The second-strand cDNA synthesis was performed using DNA Polymerase I Large (Klenow 3’-5’ exo) Fragment (New England Biolabs). All those reactions were conducted according to the manufacturer’s instructions. DNA quantification was performed using the High Sensitivity dsDNA Assay kit in a Qubit 2.0 Fluorometer (Thermo Fisher Scientific).

The libraries were constructed using the Nextera^TM^ XT - DNA Library Preparation Kit (Illumina; 6439_SW_: i503 / i701; and 6439_CM_: i504 / i701), purified with the Agencourt AMPure XP - PCR Purification (Beckman Coulter), and quantified using both High Sensitivity DNA Kits from Qubit 2.0 Fluorometer (Thermo Fisher Scientific) and 2100 Bioanalyzer (Agilent Technologies). HST was conducted by applying 2 pM of each library in the MiSeq Illumina platform using the MiSeq V3 600-cycle kit (Illumina) in paired-end mode 2 × 250 bp with a dual barcode for each sample.

*Viral metagenomic analysis* - For bioinformatics analysis, paired-end reads generated by MiSeq were submitted to an in-house analysis pipeline running on a 32-node Linux cluster using the following bioinformatics workflow: FastQC;^6^ Sickle;^7^ BWA;^8^ SAMTOOLS;^9^ and, two rounds of similarity search using BLASTx NCBI tool.^10^ Briefly, reads smaller than 50 bp were removed, and those with low sequencing quality tails were trimmed using Phred quality score 20 as the threshold. Reads were filtered using primate reference genomes, and all remaining reads were matched against an in-house viral proteome database using BLASTx with an E-value cutoff of < 10^-5^. This database was compiled using NCBI vertebrate virus reference proteome (ftp://ftp.ncbi.nih.gov/refseq/release/viral/) and the protein sequences from NCBI non-redundant FASTA file (based on annotation taxonomy in the Virus Kingdom). All assigned reads were then compared to the non-redundant protein NCBI database to remove false-positive viral hits; this database was compiled using protein sequences extracted from NCBI non-redundant FASTA files. Reference-based assembly was performed with BWA / SAMTOOLS (minimum sequencing depth for consensus calling: 10-fold), and R software was used for statistical analysis and graphic visualisation. Sequencing data files are available in the SRA database under BioProject accession PRJNA656720 and BioSample accessions: SAMN15797155 for SARS-CoV-2_6439cm library; SAMN15797156 for HSV-1_6439cm library; SAMN15797157 for SARS-CoV-2_6439sw library; and SAMN15797158 for HSV-1_6439sw library. Accession numbers for the HSV-1 and SARS-CoV-2 genome consensus sequences from the 6439_CM_ sample determined in this study are available in GenBank under the accession numbers MT876428 and MT846410, respectively.

*Phylogenetic analyses* - A dataset containing 49 genome sequences of diverse *Alphaherpesvirus* strains and the novel genome herein characterised was assembled, aligned with MAFFT v7.407,^11^ and filtered with trimAL v1.2rev59.^12^ A maximum likelihood tree was inferred from this dataset on IQ-tree^13^ using the GTR+F+G4 model.^14^ ^,^ ^15^ The Shimoidara-Hasegawa-like approximate likelihood ratio test (SH-aLRT)^16^ was used to measure branches’ statistical support. For SARS-CoV-2, the PANGOLIN web tool (https://pangolin.cog-uk.io/) was used to classify the obtained isolate. To further contextualise the novel genome, a dataset comprehending all Brazilian sequences from lineage B.1.1.33 from 2020 was assembled (*n* = 1,307; GISAID EpiCoV database accessed on 17 July 2020), and phylogenetic analysis was performed, as described above.

*Plaque reduction neutralisation test* - Plaque reduction neutralisation test (PRNT) was used to identify plaque phenotypes in the 6439_CM_ sample through neutralisation with antisera.^17^ For this, samples of known serology were used. A pre-COVID-19 sample with negative serology for HSV was used as a double-negative serum (negative for SARS-CoV-2 and HSV). A pre-COVID-19 sample with positive serology for HSV was used as an anti-HSV serum. According to the manufacturer’s instructions, HSV serology was performed using an HSV 1&2 IgG kit (DIA.PRO Diagnostic Bioprobes Srl). Anti-SARS-CoV-2 convalescent serum was obtained from a COVID-19 patient. Serology for IgG against Spike protein from SARS-CoV-2 was performed using S-UFRJ ELISA.^18^ As controls, PRNT against SARS-CoV-2 and HSV-1 isolated from sample 6439 with double-negative, anti-SARS-CoV-2, and anti-HSV sera were performed. Briefly, serum samples were incubated at 56ºC for 30 min to inactivate the complement. Two-fold serial dilutions of heat-inactivated serum were incubated with the sample 6439_CM_ containing 100 PFU of the larger plaques and 616 PFU of the smaller plaques, and, for controls, with either 100 PFU of SARS-CoV-2 or HSV-1 isolated from sample 6349 for 1 h at 37ºC to enable neutralisation to occur.

Virus-serum mixture was inoculated into confluent monolayers of Vero cells seeded in 12-well tissue culture plates (Corning). After 1 h, the inoculum was removed, and a semisolid medium (1.25% carboxymethylcellulose in alpha-MEM supplemented with 1% FBS) was added. Cells were further incubated for three days and then fixed with 4% formaldehyde solution. Viral plaques were subsequently stained for visualisation with a crystal violet solution.

*Isolation of SARS-CoV-2 from the sample 6439*_*SW*_ - To isolate SARS-CoV-2 from the sample 6439_SW_, limiting dilution was performed by a plaque assay as described above. From plaque assay results, we observed that the 1:3,000 dilution wells displayed only SARS-CoV-2 plaques. Therefore, the conditioned medium from those wells was harvested and used to inoculate Vero cells seeded in a 25 cm^2^ T-flask (8 x 10^5^ cells). After 1 h, the inoculum was discarded and DMEM supplemented with 2% FBS was added. After three days, the conditioned medium was collected, centrifuged at 300 × *g*, sterile-filtered, and stored at -80ºC. Isolation of SARS-CoV-2 from the sample 6439_SW_ was confirmed both by plaque assay and qPCR.

*Isolation of HSV-1 from the sample 6439*_*CM*_ - Isolation of HSV-1 from the sample 6439_CM_ was performed using two different techniques: neutralisation with antiserum and plaque purification.^17^

Neutralisation with antiserum was performed by PRNT, as already described. Before fixation, the conditioned medium from the well inoculated with the 1:10 anti-SARS-CoV-2 serum diluted mixed with the sample 6439_CM_ was collected and used to infect Vero cells grown in 25 cm^2^ T-flasks. Cells were incubated for three days. Then, the conditioned medium was harvested, centrifuged at 300 × *g* for 10 min, filtered through 0.22 μm membrane (Millipore), and stored at -80ºC.

For plaque purification, confluent Vero cells grown in 12-well plates were inoculated with ten-fold serial dilutions of the sample 6439_CM_. After 1 h, the inoculum was discarded, and alpha-MEM (Life Technologies) containing 0.8% low melting point agarose (Life Technologies) was added as a semisolid medium. Cells were then incubated for two days. For plaque visualisation, an additional overlay of alpha-MEM containing 0.8% agarose and 0.01% neutral red (Life Technologies) was added to the monolayers and incubated at 37ºC for 24 h. The small plaques were individually picked using sterile pipette tips, and plaque agar plugs were placed into individual tubes containing 0.4 mL DMEM. The virus stocks (200 μL) prepared from single plaques were used to infect fresh Vero cells seeded in 6-well plates, which were then incubated for three days. Conditioned medium was harvested and stored at -80ºC. Isolation of HSV-1 from the sample 6439_CM_ was confirmed both by plaque assay and PCR.

All work involving infectious SARS-CoV-2 was performed in a biosafety level (BSL)-3 containment laboratory.

*Detection of HSV-1 by PCR* - HSV-1 detection was performed using a primer set specific for HSV-1 (HSV forward: 5’-TACAACATCATCAACTTCGACTGG-3’, HSV reverse: 5’-CCTTCTTCTTGTCCTTCAGGACGG-3’) and Platinum Taq DNA Polymerase (Invitrogen), generating a 268 bp amplicon. Analysis of amplicons was performed by 1.5% gel electrophoresis. Two HSV-1 strains, acyclovir-sensitive strain (HSV-1_S_) and acyclovir-resistant strain (HSV-1_R_), were kindly provided by Dr Gabriella da Silva Mendes and Dr Norma Suely de Oliveira Santos (Instituto de Microbiologia Professor Paulo de Góes, UFRJ, Brazil) and were used as positive controls.

*Ethics* - Protocols were reviewed and approved by the National Commission for Research Ethics (CONEP, Brazil; protocol #30161620000005257; approval #3953368). An immediate relative from the deceased patient signed the informed consent form and granted permission to publish clinical data.

## RESULTS

*Patient clinical data* - A 62-year-old female patient (ID 6439) attended an emergency care unit displaying a decreased level of consciousness, disorientation, and seizures. A few days later, the patient also developed pneumonia which later evolved into respiratory distress, decreased renal function, and decompensated diabetes. Thorax computed tomography (CT) showed a pattern of diffuse infiltrate with ground-glass opacity, affecting 50% of the pulmonary parenchyma at visual analysis, typical of COVID-19, while head CT scan revealed no alterations.

**Fig. 1:**
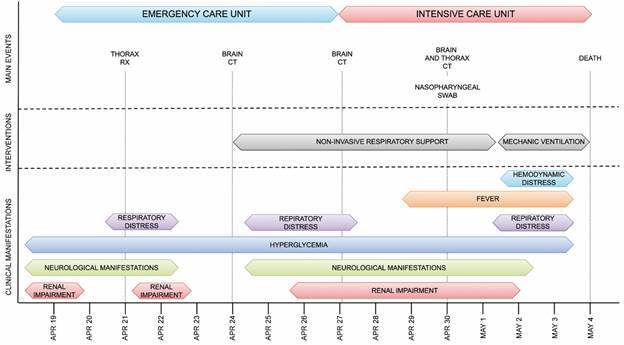
summarised timeline of the clinical evolution of patient 6439.

The patient died eight days after hospitalisation due to severe acute respiratory syndrome and COVID-19 pneumonia. Her previous medical history included systemic hypertension, insulin-dependent diabetes, oral herpes, herpetic stomatitis, previous unilateral nephrectomy, and chronic kidney disease under conservative treatment. The chronological manifestation of main symptoms and interventions is summarised in Fig. 1 and detailed in Supplementary data (Table).

*SARS-CoV-2 detection, viral load, and subgenomic RNA detection* - RT-qPCR performed with the nasopharyngeal swab sample confirmed SARS-CoV-2 infection. We noticed a remarkably low RT-qPCR Ct value for the patient’s sample (N1 Ct = 5) by the time of diagnosis, suggesting an extremely high viral load. Using a standard-curve, we estimated that the obtained Ct value from the sample corresponded to 3.64 x 10^12^ RNA copies/mL [95% confidence interval (CI): 1.08 x 10^11^-1.22 x 10^14^ copies/mL; p < 0.001]. Also, to evaluate whether this high viral load was due to a replicative virus, an RT-qPCR targeting subgenomic RNA was performed and provided evidence for viral replication (data not shown). Due to the unusually high SARS-CoV-2 viral load, we seek to better characterise the virus from this patient.

*Virus isolation and plaque analysis* - First, we performed viral isolation by inoculating Vero cells with dilutions of the filtered nasopharyngeal swab sample (6439_SW_). An unusually early cytopathic effect (CPE) was observed 24 h post-infection in the first dilutions (1:3 to 1:24). On the sixth day post-infection, all inoculated wells displayed CPE, indicating successful viral isolation. One blind passage was performed, and the conditioned medium (6439_CM_) was harvested for further analysis.

**Fig. 2:**
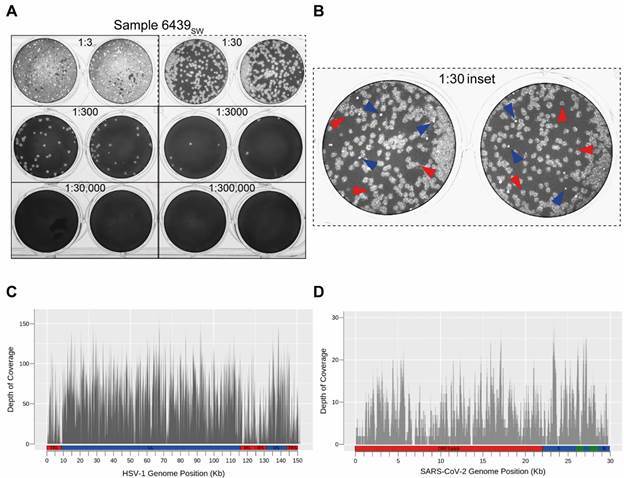
detection of viral coinfection of patient 6439. (A) Plaque assay of the nasopharyngeal swab sample from patient 6439 (6439_SW_) showing the indicated sample dilutions in duplicate wells. (B) The two different plaque phenotypes observed in this sample are highlighted in the inset of the 1:30 dilution duplicates (dashed panel). Red arrowheads correspond to the larger and turbid plaques with undefined borders, whereas blue arrowheads indicate the smaller and clearer plaques with well-defined circular borders. Depth of coverage diagram across the reference genome for Herpes simplex virus 1 (HSV-1) (C) and severe acute respiratory syndrome coronavirus 2 (SARS-CoV-2) (D) in kilobase (Kb) obtained through virome analysis. The basic structure of both genomes is indicated below the diagrams. SARS-CoV-2: non-structural coding region (red) indicated by ORF1a&b (the overlapping open reading frames 1a and 1b); and structural and accessory coding regions (blue and green) highlighting the S (spike) and N (nucleocapsid) genes. HSV-1: UL and US (blue) stand for Unique Long and Unique Short regions, respectively; and TR(L/S) and IR(L/S) (red) stand for Terminal and Internal - inverted - Repeats flanking the UL and the US regions, respectively.

Next, we performed a plaque assay to quantify infectious viral particles in 6439_SW_. Interestingly, two distinct plaque phenotypes (Fig. 2A) were observed, which were also detected in the sample 6439_CM_ [Supplementary data (Fig. 1A-B)]. These plaques differed in size, turbidity, edge definition, and abundance (Fig. 2B). Since plaque morphology mutants have been described for many viruses, including the *Coronaviridae* members Middle East Respiratory Syndrome Coronavirus (MERS)^19^ and SARS-CoV,^20^ this result suggested either the presence of two SARS-CoV-2 strains or a current productive coinfection of two different species of virus.

*High-throughput sequencing (HTS)* - Aiming to identify whether the two observed plaques were representative of two SARS-CoV-2 strains, we performed amplicon-based HTS in sample 6439_SW_. As a result, we obtained 107,537 reads mapped to the reference SARS-CoV-2 genome (isolate Wuhan-Hu-1, NCBI accession number: NC_045512.2), with a coverage of 99.88% and an average depth of 1,057.8-fold (GISAID accession number EPI_ISL_2134584). Our sequencing data neither support the hypothesis of two SARS-CoV-2 strains being present in the sample, nor did it reveal any mutations that could be associated with the high viral load (data not shown). Nevertheless, using the PANGOLIN SARS-CoV-2 genotyping tool, we were able to establish that it belongs to the lineage B.1.1.33, which is in agreement with previous data showing the circulation of this strain in Rio de Janeiro State.^21^ Moreover, no mutations characteristic of the variants of concern or interest were identified. Further phylogenetic analysis using all Brazilian SARS-CoV-2 complete genome sequences from lineage B.1.1.33 available on GISAID EpiCoV database (n = 1,307) revealed that the new characterised genome clusters as sister to a group comprehending four sequences from Rio de Janeiro and one from Paraná, though with a low support value (SH-aLTR = 0), most likely due to limited divergence among sequences [Supplementary data (Fig. 2)].

Since the presence of two different SARS-CoV-2 strains or genetic variants was discarded by genome analysis, we assessed the possibility of a productive coinfection of SARS-CoV-2 and another virus species resulting in the different plaque phenotypes observed. A metagenomic approach was pursued to accomplish this, as it simultaneously allows an unbiased and comprehensive view of the virome and the assembly of the viral genomes found.^22^

**Fig. 3:**
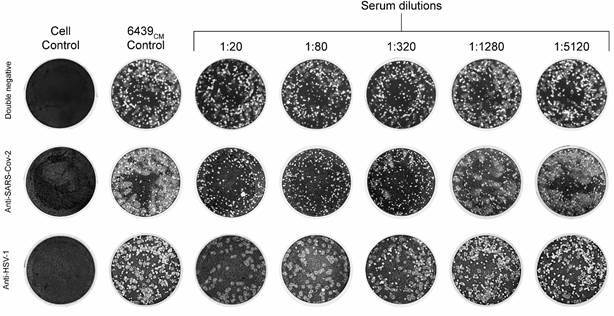
characterisation of the two plaque phenotypes. Plaque reduction neutralisation test (PRNT) using double-negative, anti-severe acute respiratory syndrome coronavirus 2 (SARS-CoV-2), anti-Herpes simplex virus 1 (HSV-1) sera. Columns correspond to fixed Vero cell monolayers subjected to the following conditions from left to right: uninfected cells (cell control), 6439_CM_ - inoculated cells without serum (virus control), and cells inoculated with 6439_CM_ pre-incubated with the indicated dilutions of the corresponding serum.

Therefore, both 6439_SW_ and 6439_CM_ were submitted to HTS, using bench protocols and *in silico* analysis aimed at viral diversity characterisation. A comprehensive analysis of raw sequencing data led to the identification of two viral families, *Coronaviridae* and *Herpesviridae*. Subsequently, reference-based genome assemblies were performed (References: SARS-CoV-2 isolate Wuhan-Hu-1, NCBI accession number NC_045512.2; HSV-1, NCBI accession number NC_001806.2), confirming the coinfection of HSV-1 and SARS-CoV-2 in both samples (Fig. 2C-D). For sample 6439_SW_, the coverage for both HSV-1 and SARS-CoV-2 was below 1%; and for sample 6439_CM_, these values correspond to 91% and 39%, respectively. Despite the low coverage reported for the sample 6439_SW_, sequencing reads were evenly distributed along the entire genomes, supporting the occurrence of both viruses in the swab sample. Comparison between genomes prior and after isolation was not possible due to the low coverage obtained for sample 6439_SW._ Phylogenetic analysis [Supplementary data (Fig. 3)] revealed that the HSV-1 (GenBank accession number: MT876428) described herein clusters within group III.^23^

*Plaque identification* - Although HTS identified both SARS-CoV-2 and HSV-1 in the sample, their respective plaque phenotypes remained unknown. To solve that, a PRNT with sera of known serology for SARS-CoV-2 and HSV was performed (Fig. 3). PRNT with anti-SARS-CoV-2 serum resulted in clearance of the larger plaques in serum dilutions from 1:10 to 1:160. Elimination of small plaques in dilutions from 1:10 to 1:40 was observed in PRNT with anti-HSV serum. PRNT of pre-COVID-19 serum from an individual with negative serology for HSV resulted in the development of both plaque phenotypes. As controls, PRNT against both SARS-CoV-2 and HSV-1 isolated from 6439 samples with double-negative, anti-SARS-CoV-2, and HSV sera was performed, and similar patterns were observed [Supplementary data (Fig. 4)]. Hence, we were able to identify SARS-CoV-2 plaques as being larger, turbid, and with undefined borders, while HSV-1 plaques were recognised as small and clear plaques with well-defined circular margins.

Through quantification of the distinct plaques, we showed that the sample 6439_SW_ contained 2.81 x 10^4^ PFU/mL of SARS-CoV-2 and 2.03 x 10^3^ PFU/mL of HSV-1 (Fig. 1A).

Isolation of SARS-CoV-2 (SARS-CoV-2^6439^) was performed by limiting dilution [Supplementary data (Fig. 5A)] and was confirmed by RT-qPCR (data not shown). To isolate HSV-1, neutralisation with anti-serum [HSV-1^6439^ PRNT; Supplementary data (Fig. 5B)] and plaque purification [HSV-1^6439^ PP; Supplementary data (Fig. 5C)] were carried out. HSV-1 virus isolation was confirmed by PCR [Supplementary data (Fig. 5D)]. Infection of Vero cells with plaque purified HSV-1 at MOI of 0.1 resulted in distinctive CPE characterised by enlarged and rounded cells, cell detachment, and cytoplasmic vacuolisation at 24 h post-infection and focal cell death, while infection at an MOI of 1 lead to pronounced cell death [Supplementary data (Fig. 5E)].

## DISCUSSION

During routine SARS-CoV-2 diagnosis, we observed a patient with an RT-qPCR Ct value of 5, which was unexpectedly low as the average Ct value within our cohort was 26.44 (range 13.01-36.98, n = 27,610 positive tests). Due to the high viral load identified in this patient, who succumbed to COVD-19 after presenting respiratory and neurological manifestations, we aimed to better characterise the infection. While there is a controversy on whether high virus load represents an increased risk for a poor COVID-19 outcome,^24^ ^,^ ^25^ ^,^ ^26^ some studies indicate that SARS-CoV-2 viral load at either diagnosis or hospital admission is associated with mortality in hospitalised patients.^27^ ^,^ ^28^ In accordance with the latter studies, the patient described in this work presented an extremely high viral load (Ct value of 5) and severe disease that quickly progressed to death.

As several SARS-CoV-2 variants of concern have been described so far and have been linked to increased transmission, virus load, and disease severity,^29^ ^,^ ^30^ we hypothesised that the virus in the sample could harbor mutations implicated in such effects, such as the variants that emerged later on 2020. However, amplicon-based HTS failed to identify any mutation known to be related to increased virus load. On the other hand, when plaque assay and a virome approach were applied, we discovered a productive coinfection between SARS-CoV-2 and HSV-1, which was further confirmed by PRNT and PCR.

Following primary infection, HSV-1 undergoes a productive infection within mucosal epithelial cells and spreads to sensory neurons, where the virus establishes latency.^31^ ^,^ ^32^ In contrast to infection in epithelial cells, which results in the release of infectious virus particles (productive infection), latently infected cells do not produce progeny virions and therefore are characterised by a nonproductive infection. Reactivation is triggered by various stimuli, including emotional stress, fever, UV exposure, immunosuppression, and local injury to tissues innervated by latently infected neurons.^33^ Reactivation from latency results in the generation of virus progeny and illness.^32^ ^,^ ^34^ Due to the patient’s clinical history of oral herpes and herpetic stomatitis, one can speculate that SARS-CoV-2 infection (either directly or through the effects of SARS-CoV-2 infection on the immune response) might have resulted in HSV-1 reactivation. However, other factors such as fever, stress, and immunosuppression may have caused or contributed to HSV-1 reactivation.

An active HSV-1 infection could also have facilitated SARS-CoV-2 infection within mucosal tissue and contributed to worsening SARS-CoV-2 prognosis. It has already been reported that coinfections may result in enhanced virus replication and virulence.^17^ Indeed, such coinfections may alter the disease severity. For instance, a study in Guinea pigs has shown that reovirus and SARS-CoV coinfection resulted in their rapid death.^35^ A study encompassing 257 COVID-19 patients found 3.1% were coinfected with HSV-1, with an increased prevalence in severe/critical cases (7%) as compared to mild ones (1.7%), although no statistical significance was reported.^36^

Moreover, given the neurological manifestations, the lack of alterations in head CT scans, and identification of productive HSV-1 and SARS-CoV-2 coinfection, we may consider viral encephalitis as a differential diagnosis. However, HSV-1 productive infection was only retrospectively diagnosed, preventing alternative therapies from being pursued in this regard. Also, despite HSV-1 being the most common causative agent of viral encephalitis,^37^ we cannot rule out SARS-CoV-2 involvement. Noteworthy, a series of encephalopathies, including encephalitis, had been associated with COVID-19.^1^ Recent findings show that SARS-CoV-2 infects neural cells, brain organoids,^38^ and the human brain.^1^ Recently, one study showed that detection of SARS-CoV-2 in post-mortem brain tissue was not associated with the severity of neuropathologic changes suggesting that neuropathogenesis might be due to an indirect effect of SARS-CoV-2 infection and might be a result of immune cell and cytokine infiltration into the CNS.^1^

Despite our efforts to characterise the coinfection reported herein, we acknowledge that several limitations prevented us from establishing a causal relation between COVID-19 severity and the coinfection, and even the dynamics of such coinfection. The limited availability of anatomically (especially from the central nervous system) and temporally diverse samples was a major hurdle, preventing us from identifying the etiological agent associated with the neurological manifestations and even determining the precise chronology of infections. This was majorly due to the rapid progression of the disease and the fact that the Brazilian public health system was overwhelmed by the pandemic. Nonetheless, by combining classical virological methods and high-end sequencing methods, we identified and characterised the SARS-CoV-2 and HSV-1 productive coinfection. Although SARS-CoV-2 and HSV-1 coinfection has been previously reported,^36^ ^,^ ^39^ ^,^ ^40^ this study shows the production of HSV-1 infectious particles, differentiating it from latent infection. Thus, while we report a single patient’s case, this study acts as a warning to address the presence of productive HSV-1 coinfection in a significant number of severe COVID-19 cases, particularly those with neurological manifestations that lack corresponding alterations in brain CT scans. On the other hand, this study also serves as an alert to the possibility of reactivation of HSV-1 due to SARS-CoV-2 infection, which may be associated with a deterioration of the patient’s condition.

Here, we identified and characterised SARS-CoV-2 and HSV-1 from a productive coinfection in a patient with an unusually high SARS-CoV-2 load and who rapidly evolved to death. Although we cannot establish a causal relationship between the coinfection and the severity of the case, the high worldwide HSV-1 prevalence (66.6%) among individuals aged 0-40 years^41^ highlights the relevance of investigating SARS-CoV-2 and HSV-1 coinfection. As mentioned above, despite our best efforts to characterise the coinfection, several aspects regarding its chronology and the interplay between viruses worsening the disease progression remain elusive. Therefore, further studies are necessary to address the role of SARS-CoV-2 in HSV-1 reactivation and the consequences of this productive coinfection in clinical outcomes.
