# Supplementary material for: Identification and characterisation of SARS-CoV-2 and Human alphaherpesvirus 1 from a productive coinfection in a fatal COVID-19 case
Source: Mem Inst Oswaldo Cruz. 2022 Jan 10;116:e210176. doi: 10.1590/0074-02760210176 (PMC8752051; doi:10.1590/0074-02760210176)
Supplement: Supplementary file 1 [file 1678-8060-mioc-116-e210176-s.pdf]

## Covid19-UFRJ Workgroup

The COVID19-UFRJ Workgroup encompasses all individuals (physicians, professors, technicians, postgraduate and undergraduate students, and volunteers) who participated in the COVID-19 diagnostics at the Universidade Federal do Rio de Janeiro during the COVID-19 pandemic.

## Members

Átila Duque Rossi, Bruno Clarkson, Camila de Almeida Velozo, Camila Nacif, Camille Victória, Leal Correia de Silva, Caroline Macedo Nascimento, Carolyne Lalucha Alves L da Graça, Cassia Cristina Alves Gonçalves, Címtia Policarpo, Cynthia Chester Cardoso, Débora Souza Faffe, Ekaterini Goudouris, Elaine Sobral da Costa, Elisangela Costa da Silva, Érica Ramos dos Santos Nascimento, Fabio Hecht Castro Medeiros, Fernanda Leitão dos Santos, Fernando Luz de Castro, Francine Bittencourt Schiffler, Gabriela Bergiante Kraychete, Guilherme Sant'Anna de Lira, Gustavo Peixoto Duarte da Silva, Helena Keito Toma, Huang Ling Fang, Inês Corrêa Gonçalves, Ingrid Camelo da Silva, Isabela de Carvalho Leitão, Isabela Labarba Carvalho de Almeida, Jessica Maciel de Almeida, Joissy Aprigio de Oliveira, Juliana Cazarin de Menezes, Juliana Tiemi Sato Fortuna, Karyne Ferreira Monteiro, Lendel Correia da Costa, Letícia Averbug Corrêa, Liane de Jesus Ribeiro, Lídia Theodoro Boullosa, Liliane Tavares de Faria Cavalcante, Lucas Matos Millioni, Manoel Itamar do Nascimento, Marcelo Amaral de Souza, Marcelo Calado de Paula Tôrres, Mariane Talon de Menezes, Marisa Souza Correia, Mateus Rodrigues de Queiroz, Matheus Augusto Calvano Cosentino, Mayla Gabryele Miranda de Melo, Pedro Henrique Costa da Paz, Pedro Henrique Costa da Paz, Richard Araujo Maia, Rodrigo de Moraes Brindeiro, Sérgio Machado Lisboa, Thamiris dos Santos Miranda, Victor Akira Ota and Viviane Guimarães Gomes.

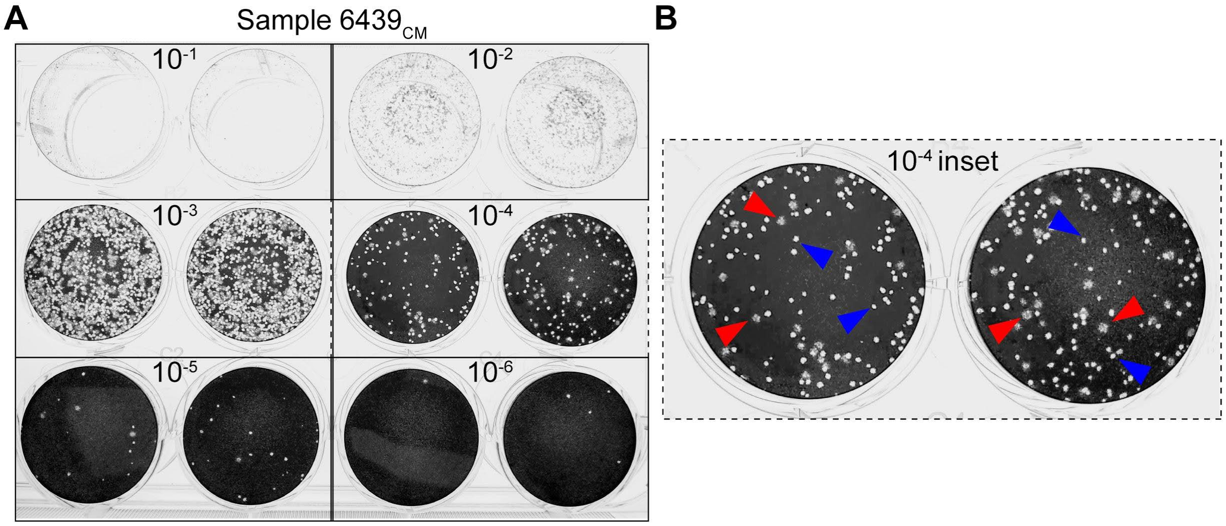


Fig. 1: plaque assay plate of the sample $6439_{CM}$ (A), showing the indicated inoculum dilutions in duplicate wells of infected Vero cell monolayers. The two plaque phenotypes observed in this sample are highlighted in the inset (B) of the $10^{-4}$ dilution duplicates (dashed panel). Red arrowheads correspond to the larger and turbid plaques with undefined borders, whereas blue arrowheads indicate the smaller and clearer plaques with well-defined circular borders.

cc BY

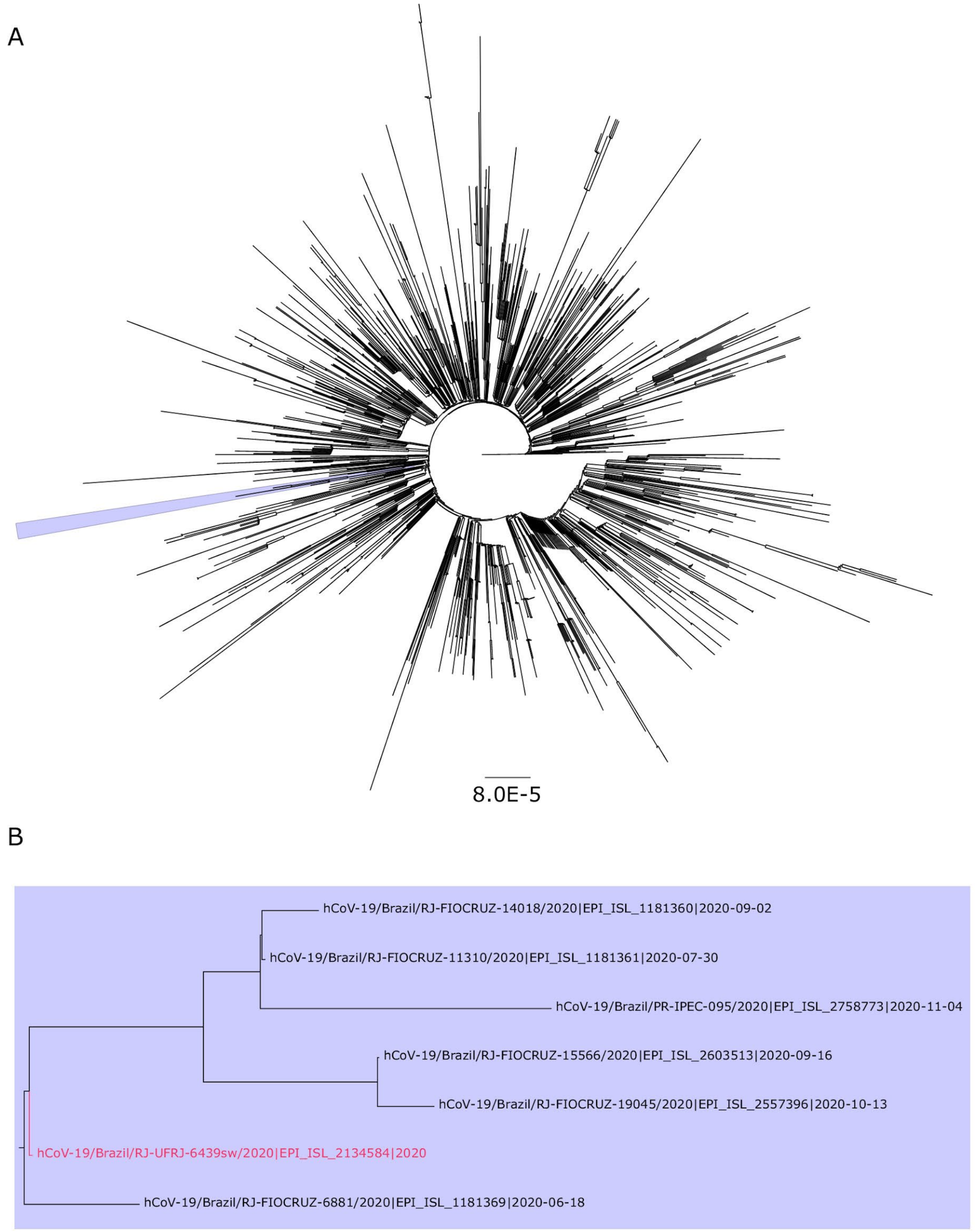


Fig. 2: phylogenetic characterisation of severe acute respiratory syndrome coronavirus 2 (SARS-CoV-2) obtained from the sample $6439_{sw}$. (A) Maximum likelihood phylogenetic tree inferred from a dataset comprehending all Brazilian SARS-CoV-2 complete genome sequences from lineage B.1.1.33 available on GISAID EpiCoV database (as of July 17, 2021) ($n$ = 1,307). The light purple shade highlights the clade where the novel genome clustered. (B) Zoom on the previous clade, revealing that the new characterised genome clusters as sister to a group comprehending four sequences from Rio de Janeiro and one from Paraná, though with low support value (SH-aLRT = 0) to the overall tree due to limited sequences divergence. The tree was arbitrarily rooted on the oldest sequence available in the dataset for visualisation purposes only. The scale bar indicates average substitutions per site.

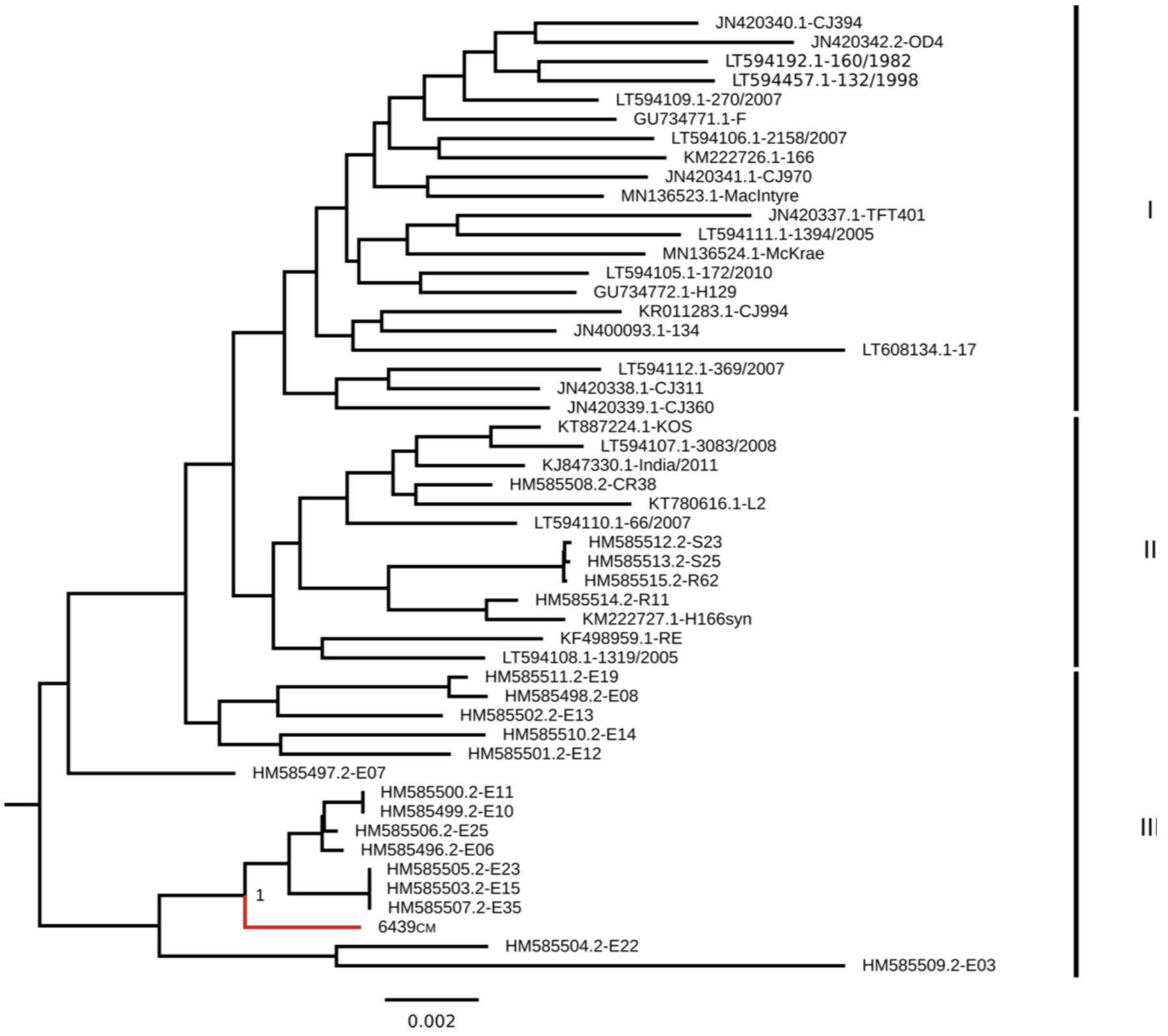


Fig. 3: maximum likelihood phylogeny containing the currently characterised diversity of alphaherpesviruses. The tree indicates that the HSV-1 strain herein described ($6439_{CM}$) clusters with maximum support as a sister to a clade composed of strains E11, E10, E25, E6, E23, E15, and E35. Black bars mark *Alphaherpesvirus* groups described in Pffaf et al.,[1] and the red branch indicates the HSV-1 sequence obtained from sample $6439_{CM}$. The tree was midpoint rooted for visualisation purposes only. The scale bar indicates nucleotide substitutions per site.

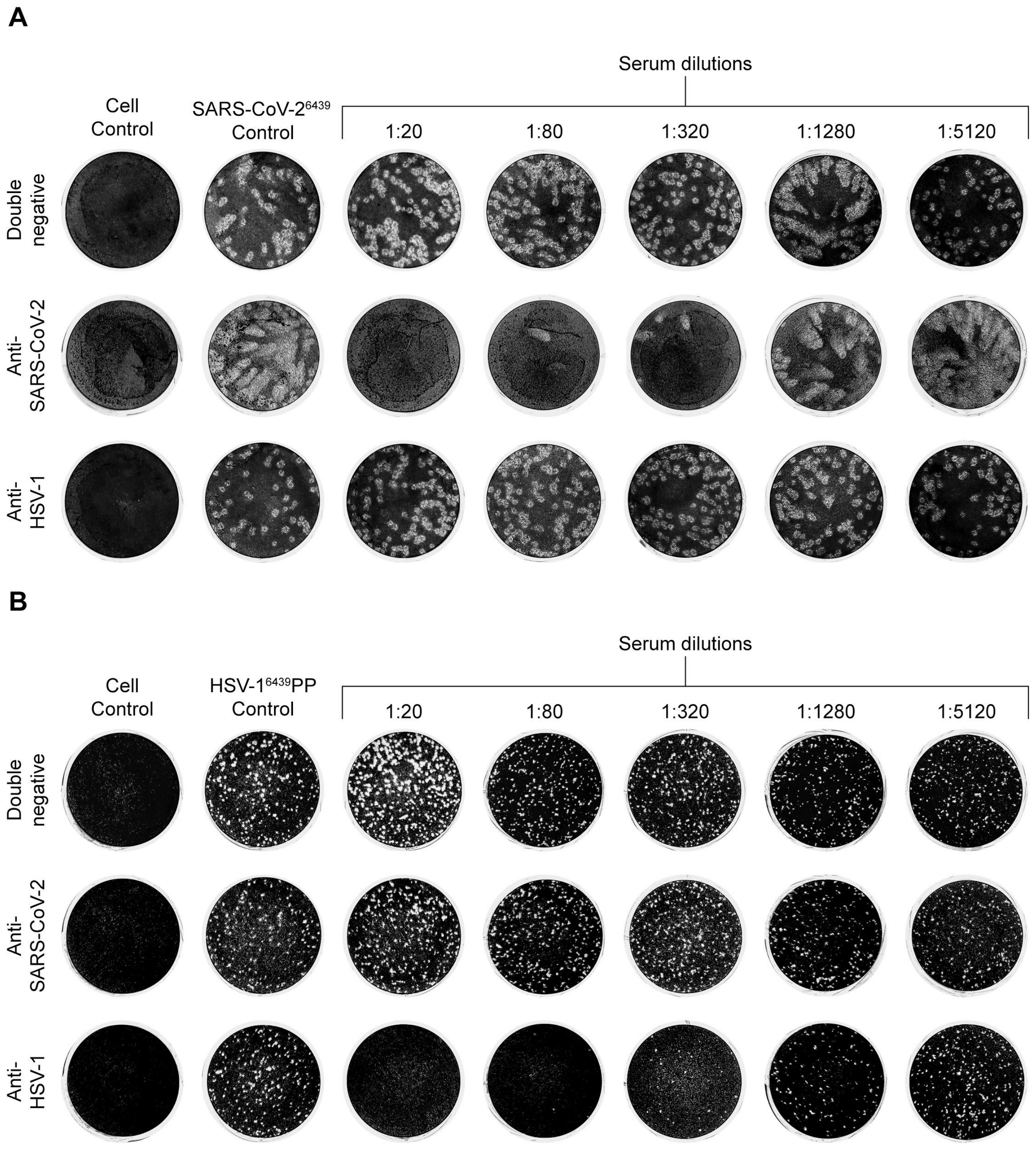


Fig. 4: plaque reduction neutralisation test (PRNT) with SARS-CoV-2$^{6439}$ (A) and HSV-1$^{6439}$ PP (B), the isolated viruses from 6439 samples using double-negative, anti-SARS-CoV-2, and anti-HSV sera. Columns correspond to fixed Vero cell monolayers subjected to the following conditions from left to right: uninfected cells (cell control), virus inoculated cells without serum (virus control), and cells inoculated with 100 PFU of virus pre-incubated with the indicated dilutions of the corresponding serum.

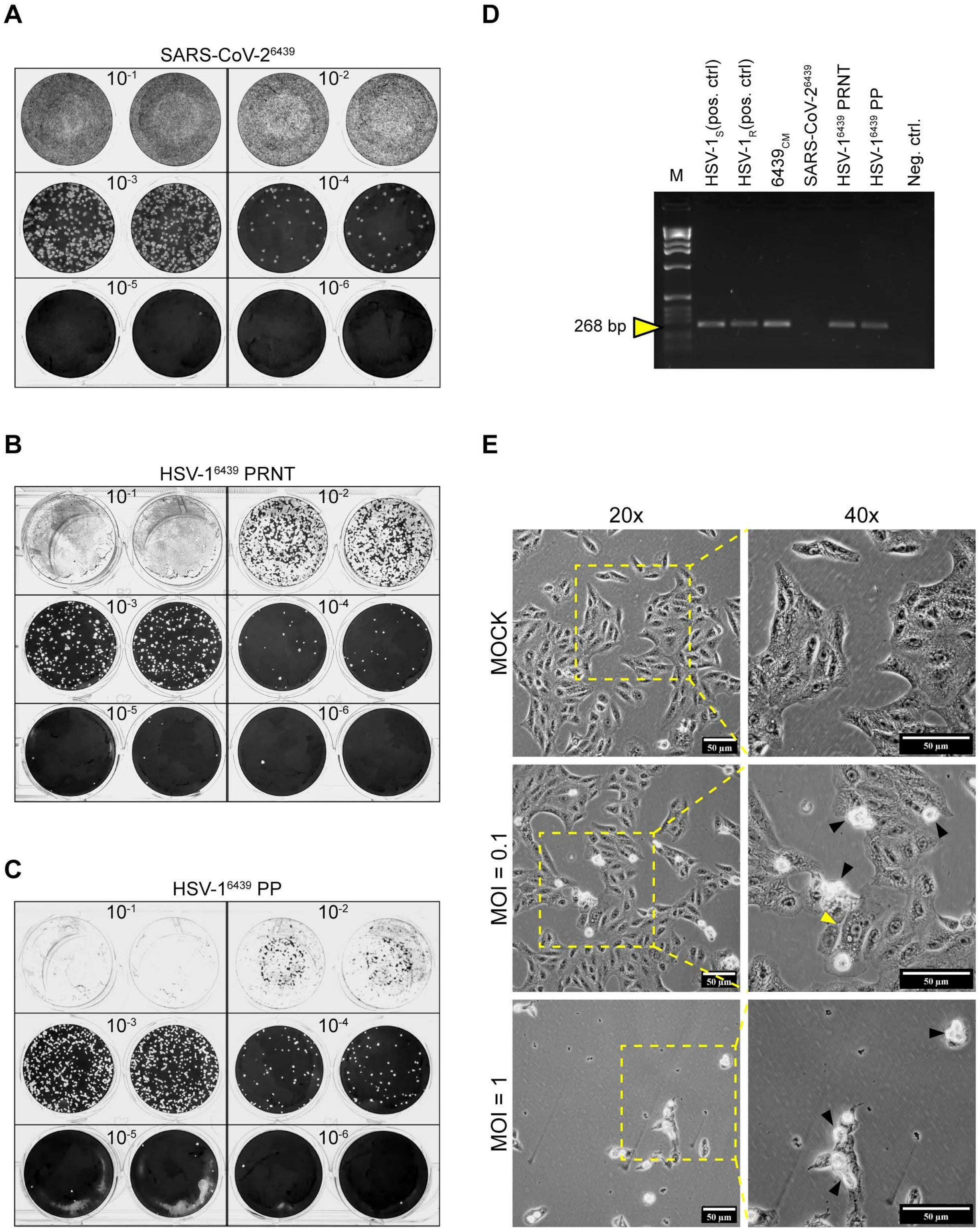


Fig. 5: plaque assay plates of severe acute respiratory syndrome coronavirus 2 (SARS-CoV-2)[6439](A), Herpes simplex virus 1 (HSV-1)[6439] plaque reduction neutralisation test (PRNT) (B), HSV-1[6439] PP (C). Plates showing the indicated inoculum dilutions in duplicate wells of infected Vero cell monolayers. (D) HSV-1 detection was performed by polymerase chain reaction (PCR). Amplicons (268 bp) were visualised in 1.5% agarose gel electrophoresis. Lane M: 1 kb Plus DNA Ladder (Invitrogen); lanes 2 and 3: HSV-1 positive controls; lane 8: no template control. (E) Microscopy of Vero cells infected with purified HSV-1 from the sample $6439_{CM}$ at MOI of 0.1 and 1 after 24 hpi. Panels on the right are magnifications of the correspondent yellow dashed inset. Black arrowheads indicate enlarged and round dying cells, and yellow arrowheads indicate cytoplasmic vacuolisation. White scale bars = 50 µm.

TABLE

The clinical course of patient 6439

| Day | Date | Events | Symptoms/Clinical signs | Laboratory findings | Treatments |
|---|---|---|---|---|---|
| 1 | April 19 | Presented at emergency care unit (ECU) where hospitalisation was requested for decompensated insulin-dependent diabetes mellitus | Decreased level of consciousness. | Ht-37.4%; WBC-10,500/mL (segmented neutrophils-45%, band forms-3%, lymphocytes-41%); blood urea-146mg/dL; creatinine-3.79mg/dl; elevated blood glucose | IV hydration, insulin |
| 2 | April 20 | ECU | Decreased level of consciousness, Glasgow 9, eupneic in ambient air, dehydrated, physical examination without other significant changes. | Ht-41.4%, WBC-20,900/ml (band forms-8%) | eftriaxone, clindamycin, insulin |
| 3 | April 21 | ECU, chest X-ray | Disoriented, eupneic in ambient air, reentrant seizures | Chest X-ray: right perihilar pulmonary infiltrate | Meropenem, vancomycin, hydantal, insulin |
| 4 | April 22 | ECU | Aphasic, unresponsive, afebrile, pulmonary rhonchi and rales; $SpO_2$:92% in ambient air; | Ht-41.4%, WBC-23,200/mL (band forms-10%); urea-176mg/dl; creatinine-5,2mg/dl; HGT-141-489mg/dl | Meropenem, vancomycin, hydantal, insulin |
| 5 | April 23 | ECU | Not reported | Ht-41.4%, WBC-22,700/mL (band forms-10%); blood glucose-155mg/dl | Meropenem, vancomycin, hydantal, insulin |
| 6 | April 24 | ECU, 1st Head CT | Head CT | WBC-13,700/mL (band forms-8%); urea- 142mg/dl; creatinine-4,5mg/dl. Head CT without alterations | Non-invasive oxygen support, meropenem, vancomycin, hydantal, insulin |
| 7 | April 25 | ECU | Disoriented, seizures, afebrile, pneumonia, uncontrolled hypertension, no other major changes | Not reported | Non-invasive oxygen support, meropenem, vancomycin, hydantal, insulin, Nipride® |
| 8 | April 26 | ECU | Disoriented but responsive, eupneic under nasal oxygen catheter, afebrile. | Ht-34,2%, WBC-17,300/mL (band forms-10%); urea- 214mg/dl, creatinine-6,2mg/dl; blood glucose-312mg/dl | Non-invasive oxygen support, meropenem, vancomycin, hydantal, insulin, Nipride® |
| 9 | April 27 | 2nd Head CT and transfer to HFSE intensive care unit (ICU) for cerebrovascular accident (CVA) and pneumonia | Afebrile at admission, respiratory distress, Glasgow 13, left hemiparesis, dysarthria, sacral pressure ulcer, worsening renal function, decompensated diabetes. | No alterations in head CT. | Zero diet, nasogastric catheter, non-invasive oxygen support, meropenem, vancomycin, hydantal, insulin, losartan, amlodipine, simvastatin, clexane |
| 10 | April 28 | ICU, Hemodialysis started | No major evolutive changes | Ht-30,4%; WBC-13,700/mL; urea-217mg/dl, creatinine-5,7mg/dl, blood glucose-234mg/d; serum C-reactive protein-10.2mg/dl | Enteral diet, non-invasive oxygen support, meropenem, vancomycin, hydantal, insulin, losartan, amlodipine, simvastatin, clexane, intermittent daily hemodialysis |
| 11 | April 29 | ICU | Febrile (38ºC) | Ht-27%; WBC-10,100/mL; urea-110mg/dl; creatinine-3,0mg/dl; blood glucose-249mg/d; serum C-reactive protein-12.9mg/dl. | Enteral diet, non-invasive oxygen support, meropenem, vancomycin, hydantal, insulin, losartan, amlodipine, simvastatin, clexane, intermittent daily hemodialysis |
| 12 | April 30 | ICU, nasopharyngeal swab, head and thorax CT, notification to epidemiological surveillance | Disoriented, febrile (37.2-38.6ºC) | Ht-29.2%; WBC-14,300/mL; urea- 86/dl, creatinine-3,0mg/dl, blood glucose-126mg/dl, serum C-reactive protein-20.1mg/dl. Thorax CT with typical COVID-19 infiltrate. No alterations in head CT. | Enteral diet, non-invasive oxygen support, meropenem, vancomycin, hydantal, insulin, losartan, amlodipine, simvastatin, clexane, ivermectin, intermittent daily hemodialysis |
| 13 | May 1 | ICU | 37.5°C; no major evolutive changes. | Ht-30% WBC-16,300/mL; urea-113/dl, creatinine-3,8mg/dl, blood glucose(plasma)-172mg/dl, serum C-reactive protein-20.1mg/dl | Enteral diet, non-invasive oxygen support, meropenem, vancomycin, hydantal, insulin, losartan, amlodipine, simvastatin, clexane, ivermectin, intermittent daily hemodialysis |
| 14 | May 2 | ICU, Orotracheal intubation, and mechanical ventilatory support | Desaturation, arterial hypotension, torpor, afebrile | HGT:150-253mg?dL; serum C-reactive protein-21.6mg/dl | Invasive mechanical ventilation, enteral diet, meropenem, vancomycin, hydantal, simvastatin, clexane, insulin, noradrenaline, intermittent daily hemodialysis |
| 15 | May 3 | ICU, Mechanical ventilatory support | Febrile-38.3°C, hemodynamic instability | | Invasive mechanical ventilation, enteral diet, meropenem, vancomycin, hydantal, simvastatin, clexane, insulin, noradrenaline, intermittent daily hemodialysis |
| 16 | May 4 | Death | Refractory shock and death at 6:15 am. Death declaration: Part I. Severe acute respiratory syndrome, COVID-19 pneumonia. Part II CVA - bronchoaspiration | | |

]ECU: emergency care unit; ICU: intensive care unit; CT: computed tomography; HSFE: Hospital Federal dos Servidores do Estado do Rio de Janeiro; CVA: cerebrovascular accident; Ht: blood hematocrit; WBC: white blood count; HGT: hemoglucotest; IV: intravenous.

## REFERENCE

1. Pfaff F, Groth M, Sauerbrei A, Zell R. Genotyping of herpes simplex virus type 1 by whole-genome sequencing. J Gen Virol. 2016; 97(10): 2732-41.
